# Optimization of Laser Based-Powder Bed Fusion Parameters for Controlled Porosity in Titanium Alloy Components

**DOI:** 10.3390/ma17225572

**Published:** 2024-11-14

**Authors:** Emanuele Vaglio, Federico Scalzo, Marco Sortino, Giovanni Totis, Roberto Cremonese, Massimiliano Boccia, Maila Danielis

**Affiliations:** 1 Polytechnic Department of Engineering and Architecture, University of Udine, Via delle Scienze 206, 33100 Udine, Italy; federico.scalzo@uniud.it (F.S.); marco.sortino@uniud.it (M.S.); giovanni.totis@uniud.it (G.T.); 2 CTS H2 s.r.l., viale Lino Zanussi 1, 33070 Brugnera, Italy; r.cremonese@ctsh2.com (R.C.); m.boccia@ctsh2.com (M.B.)

**Keywords:** laser based-powder bed fusion, process parameters optimization, titanium alloy, controlled porosity, surface area

## Abstract

Laser based-powder bed fusion (LB-PBF) enables fast, efficient, and cost-effective production of high-performing products. While advanced functionalities are often derived from geometric complexity, the capability to tailor material properties also offers significant opportunities for technical innovation across many fields. This study explores the optimization of the LB-PBF process parameters for producing Ti6Al4V titanium alloy parts with controlled porosity. To this end, cuboid and lamellar samples were fabricated by systematically varying laser power, hatch distance, and layer thickness according to a full factorial Design of Experiments, and the resulting specimens were thoroughly characterized by analyzing envelope porosity, surface roughness and waviness, surface morphology, and surface area. A selection of specimens was further examined using small-angle X-ray scattering (SAXS) and wide-angle X-ray scattering (WAXS) to investigate the atomic structure and nanometric porosity of the material. The results demonstrated the possibility to finely control the porosity and surface characteristics of Ti6Al4V within specific LB-PBF process ranges. The pores were found to be mostly closed even for thin walls, while the surface roughness was recognized as the primary factor impacting the surface area. The lamellar samples obtained by exposing single scan tracks showed nearly an order-of-magnitude increase in both surface area and pore volume, thereby laying the groundwork for the production of parts with optimized porosity.

## 1. Introduction

In recent years, additive manufacturing (AM) has experienced significant growth due to its ability to create complex geometries, accelerate product development, and enhance production sustainability [1].

Among the various AM technologies, laser based-powder bed fusion (LB-PBF) is widely recognized as the leading method for producing high-quality industrial components [2]. In this process, metal powder is spread in thin layers and selectively melted by a laser. This approach allows to effectively produce highly complex, near-net-shape geometries, overcoming the limitations of traditional manufacturing processes [3]. Concurrently, LB-PBF enhances production efficiency by minimizing the need for extensive process design and setup times, as well as by reducing lengthy post-processing steps [4], and eliminating the need for expensive specialized tools or equipment [5]. Furthermore, this method facilitates the integration of components into monolithic structures, thereby reducing assembly requirements and maintenance needs, while promoting waste reduction through efficient powder usage and recycling [5].

Traditionally, LB-PBF has been predominantly employed in the biomedical and aerospace fields [6]. Major applications in these areas arose from the capability to produce topologically optimized geometries with complex beam-like features [7] and reticular structures enabling enhanced mechanical [8] and biological behavior [9]. Subsequently, its application has progressively expanded to other strategic sectors, including automotive [10], naval [11], energy [12], and electrical sectors [13], as well as manufacturing for the production of tools and equipment [14,15]. Nevertheless, there are still many other potential applications, which are currently being investigated.

While novel AM products are generally based on research into geometric complexity, the capability to tailor material properties also provides substantial opportunities for technical innovation. Specifically, porosity modulation has considerable applicative potential in structural mechanics for the development of topologically optimized components, in biomedical engineering for the production of implants and prosthetics, in energy engineering for the fabrication of fuel production and storage devices, and in chemical engineering for the production of filtration and catalytic systems. In this context, structured parts in the form of honeycombs or periodic open cellular structures (POCSs) are gaining particular attention. These structures are a distinct subset of lattice structures composed of periodic unit cells that can be engineered to optimize the mechanical [16] and thermo-hydraulic performance [17].

POCSs can be efficiently produced using LB-PBF by combining multiple thin walls to form open channels ranging from millimeters to decimeters in size. At the end of the process, the part can be separated from the building platform by using low-force methods, such as Electrical Discharge Machining, and the inner unmelted powder can be easily removed with either suction or by simply washing the product. However, strict control over material porosity is essential for producing structured parts [18,19,20] where high cellular porosity combined with low internal porosity is required to achieve both high specific surface area and good mechanical resistance. In this framework, the high surface area of POCSs, as well as that of other lattice structures and bulk parts, can be further enhanced by controlling surface porosity. This concept is pushed to the limit in structured catalysts, where increasing the specific surface area (SSA) of complex channel geometries allows to overcome traditional mass and heat transfer limitations [21] while simultaneously increasing the gas/solid interface and promoting catalytic reactions, hence resulting in more efficient processes [22,23,24,25]. Consequently, periodic open cellular structures are regarded as the ideal reference area for investigating and applying porosity control, yet similar benefits can also be harnessed in many other fields mentioned above, where more complex lattice structures or even bulk parts are used. However, the potential of LB-PBF to produce materials with controlled porosity is still largely unexplored.

Porosity is an intrinsic characteristic of the LB-PBF process, primarily arising from insufficient powder melting or gas entrapment [26]. Pore sizes typically range from 10 μm to 100 μm in diameter, and from 1.9 × 10^4^ μm^3^ to 9.52 × 10^5^ μm^3^ in volume. Insufficient melting pores generally form between molten tracks and vary widely in size, while gas pores tend to form within the molten tracks [27]. The coalescence of different pore types often leads to microcrack formation [28,29]. In general, porosity is more pronounced in thin-walled structures compared to solid parts due to edge effects [30].

Insufficient melting is primarily attributed to inadequate overlap between molten tracks or excessive layer thickness, resulting in horizontally elongated pores in the former and vertically elongated pores in the latter [31]. Nevertheless, also very low scan speeds, excessive laser power, or beam defocusing can result in irregularly shaped pores due to insufficient melting [30].

In contrast, gas porosity is typically induced by excessive laser power or insufficient scan speeds, which can trigger the keyhole phenomenon, particularly at the end of molten tracks where abrupt laser shut-off occurs [30]. Keyhole porosity can be mitigated by dynamically adjusting laser power based on scan speed to maintain consistent power density throughout the process [32], or by increasing scan speed to control the molten pool dynamics and enhance cavity stability [33]. Notably, peculiar recent studies have shown that also incorporating nanoparticles into the raw material reduces keyhole porosity by increasing the viscosity of the molten pool [34].

Overall, reducing energy density can decrease gas-induced porosity but may simultaneously increase porosity from insufficient melting, potentially leading to a net increase in material porosity [28]. Abele et al. [35] demonstrated that optimizing laser power, scan speed, and track spacing allows control of the porosity of 17-4 PH stainless steel within a range of 0.99–17.35%. The obtained average pore sizes ranged from 7 μm to 16 μm, with a permeability coefficient of 2560 × 10^−12^ m^2^ measured in a 250 μm thick wall. The study also highlighted a near-linear relationship between porosity and the material’s mechanical properties.

Although many studies have examined the porosity of materials produced by LB-PBF, limited efforts have been made to control it through process parameter optimization. In most research works, the objective was instead the opposite: minimizing porosity by primarily controlling the melt pool dynamics and geometry [36] to avoid stress concentration [37], prevent the reduction in the load-bearing cross-sectional area [38], and ultimately preserve the mechanical properties [39]. To this end, process maps that identify low-porosity process windows are typically created by experimentally varying individual process parameters or by using dimensionless indices such as energy density. Analytical and numerical models were also developed to support this effort [26], while real-time monitoring and control systems can be implemented to regulate the process within a safe operational window [40]. However, these approaches do not allow for consistently achieving a target-design porosity. Consequently, the current understanding of porosity control in LB-PBF remains insufficient, underscoring the need for further scientific investigation to advance technological progress in this area.

This paper explores the optimization of the LB-PBF parameters to enable the fabrication of Ti6Al4V alloy parts and POCSs with controlled internal and surface porosity by providing analytical equations relating material porosity to process parameters, and thoroughly describing the fundamental origins of pores and their qualitative characteristics. The Ti6Al4V alloy is renowned for its high strength-to-weight ratio [41], excellent corrosion resistance [42], outstanding biocompatibility [43], and minimal susceptibility to radioisotope transmutation [44]. These attributes make it an ideal choice for advanced applications in the aforementioned industries. Additionally, its high strength may provide superior durability compared to other materials even at high porosity levels, while its excellent processability facilitates the efficient production of high-quality components with reduced processing time and minimal finishing work. Overall, these qualities make the selected material the ideal candidate for producing parts with controlled porosity across various fields and applications.

For the purposes of this research, both cuboid and lamellar samples were produced by systematically varying the processing conditions, and the samples were characterized through porosity analysis, surface area measurements, and optical microscopy. Additionally, selected specimens underwent small-angle X-ray scattering (SAXS) and wide-angle X-ray scattering (WAXS) analysis to evaluate their atomic structure nanometric porosity. The findings provided unprecedented data of primary scientific importance and proved that both the porosity and surface characteristics of Ti6Al4V can be finely regulated by selecting process parameters within a well-defined but narrow region, thus yielding useful guidelines for the production of porous parts.

## 2. Materials and Methods

**Figure 1:**
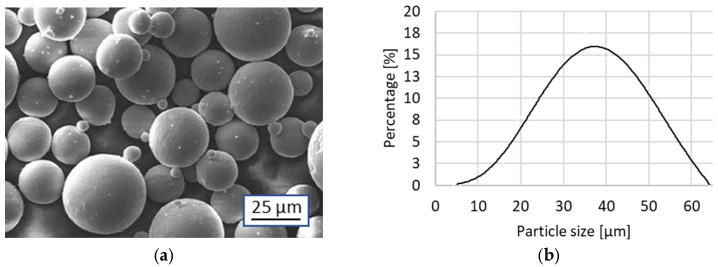
(**a**) Scanning electron microscope view of the Ti6Al4V powder, and (**b**) particle size distribution.

The experiments were conducted using commercially sourced Ti6Al4V powder. The raw material consisted of spherical particles ranging from 18 μm (10th percentile) to 50 μm (90th percentile) in diameter, with a median of 32 μm (Figure 1). The chemical composition of the material is provided in Table 1.

A total of 18 cuboid samples measuring 15 × 5 × 5 mm were produced by varying the process parameters according to a full factorial Design of Experiments. The cross-section size was chosen to simulate a single element in the typical island strategy, while the experimental design was established starting from optimal process conditions [45]. The factors under investigation included laser power, which can lead to pore formation owing to insufficient melting or gas entrapment; layer thickness, which can cause interlayer porosity; and hatch distance, which may result in intertrack porosity [46]. The specific levels were selected to promote the formation of insufficient melting pores, as gas pores are generally too large and sparse to be suitable for the intended application. In contrast, scan speed and laser beam size were kept constant, as they were assumed to be redundant compared to the other parameters for controlling porosity and are associated with other LB-PBF phenomena, such as balling and keyhole [47]. The specific combinations of process parameters are listed in Table 2 along with the sample identification numbers and optimal reference parameters.

All samples were produced using a Concept Laser M2 Cusing machine equipped with an ytterbium-doped fiber laser. The fabrication process was carried out in an inert argon atmosphere, with residual oxygen levels maintained below 0.5%. The laser path followed a bidirectional pattern which was rotated by 90° with each layer. To isolate the effects of the tested parameters, no contour scanning was performed. The samples were positioned near the process gas suction duct to facilitate the extraction of melting fumes and were staggered to minimize the risk of detrimental interactions. Additionally, the samples were rotated by 45° relative to the recoating direction to ensure optimal powder deposition.

**Figure 2:**
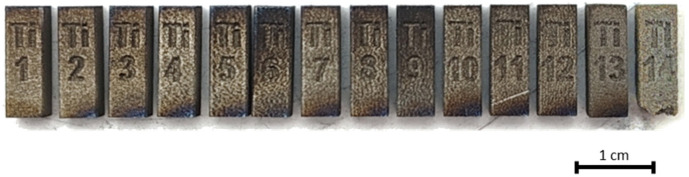
Final cuboid samples fabricated from Ti6Al4V alloy.

The samples were then heat-treated to eliminate residual thermal stresses and to simulate real operating conditions. The process involved heating to 840 °C over 4 h, holding at that temperature for 2 h, and slowly cooling to 150 °C in a closed furnace. Finally, the samples were separated from the build platform using wire electrical discharge machining. The obtained samples are illustrated in Figure 2.

To investigate the effect of process parameters on material properties, the envelope density of the cuboid samples was first calculated using a Mitutoyo Digimatic Absolute AOS digital caliper and a Sartorius A200S analytical scale.

Subsequently, the surface characteristics were analyzed using a Sensofar S neox Five Axis 3D confocal microscope. The scanning parameters were optimized by adjusting the objective lens at three magnification levels, the light intensity, and the detector sensitivity, while maintaining a constant 35% overlap rate between the fields of view and scanning in continuous mode. The primary optimization criteria included the point acquisition rate, defined as the ratio of measured points to the total number of pixels, and the differences in average linear roughness (R_ac_) along the sample growth direction across the objectives, with the 20× objective serving as the accuracy reference.

The analysis revealed that the 10× objective reduced scanning times by approximately 20 times compared to the 20× objective, while maintaining a high point acquisition rate and minimal differences in roughness measurements. In contrast, the 5× objective demonstrated excessive inaccuracy. Consequently, all the samples were analyzed using the Nikon T Plan SLWD 10×/0.20 objective, with maximum brightness and detector sensitivity, 35% overlap between the fields of view, and continuous scanning mode. The accuracy of the obtained measurements exceeded 600 nm (with a 95% confidence level), while the repeatability was better than 20 nm [48].

The collected data were processed with a mild noise reduction filter and used to extract images and profiles for analyzing surface morphology, roughness, and waviness in both parallel and perpendicular directions with respect to the sample build direction. Average roughness and average waviness were calculated according to ISO 4287 [49] standards, using a cut-off wavelength of $\lambda_{cut-off}=0.8\;mm$ [50].

Based on the obtained results, the most promising parameters for producing porous parts were selected to fabricate lamellar samples for surface area measurements, small-angle X-ray scattering (SAXS), and wide-angle X-ray scattering (WAXS) analysis. The selected parameters are detailed in Table 2 and correspond to those yielding the highest porosity among the well-formed cuboids.

**Figure 3:**
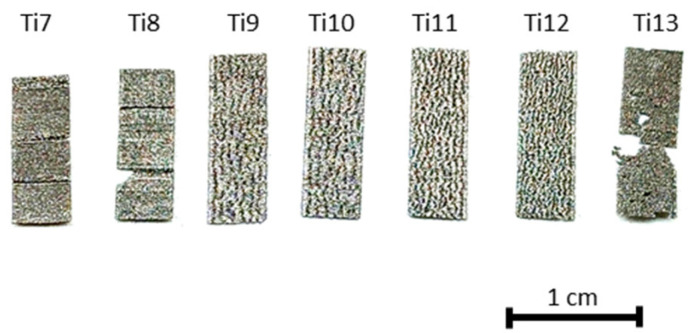
Representative examples of the final lamellar samples fabricated from Ti6Al4V alloy.

A total of 35 lamellar samples (5 repetitions per type) measuring 15 × 5 × 0.1 mm were produced with the longest side parallel to the building direction. To this end, the same machine and conditions used for the cuboid samples were employed, except for the scan strategy, which in this case consisted in exposing a single 5 mm track per layer. The same thermal treatment was also applied. One representative example of each type of the obtained samples is illustrated in Figure 3.

Surface area and pore size measurements were obtained using an Anton Paar NOVA 800 analyzer. Physisorption isotherms were measured at 77 K using N_2_ as probe gas, allowing to probe micro-, meso-, and macropores within the 1.1–500 nm size range. Prior to the analysis, samples were outgassed at 300 °C in vacuum for 3 h to remove surface adsorbates from the production process. Identical measurements were also conducted on cuboid samples for comparative analysis. Collected data were used to calculate surface area and pore distribution curves using single-point BET analysis on the second adsorption point and the BHJ method on the desorption branch, respectively [51].

Finally, small-angle X-ray scattering (SAXS) and wide-angle X-ray scattering (WAXS) analyses were conducted on the most promising Ti9 sample using synchrotron light at an energy of 16 keV. SAXS and WAXS are techniques used to analyze the structural properties of materials at different length scales. SAXS is specifically designed to study structures in the range of 1–100 nm and provides valuable information about the overall shape, size, and distribution of particles, pores, or domains within a material. WAXS focuses instead on structures in the range of 0.1–1 nm and offers insights into crystalline structures, including atomic and molecular arrangements, lattice parameters, and atomic spacing. These valuable data are very rare and complete this comprehensive investigation into the porosity of Ti6Al4V produced by LB-PBF.

## 3. Results and Discussion

Most of the tested process parameters resulted in well-formed, conformal cuboid samples. Ti1–Ti9 showed no visible signs of porosity, Ti10–Ti13 appeared visibly altered, and Ti14 had a strong tendency to crumble even under minimal stress. Ti15–Ti18 failed to form due to insufficient energy input. It can be concluded that the experimental design effectively investigated the entire process spectrum, from minimal porosity to complete particle aggregation failure.

### 3.1. Envelope Porosity

**Figure 4:**
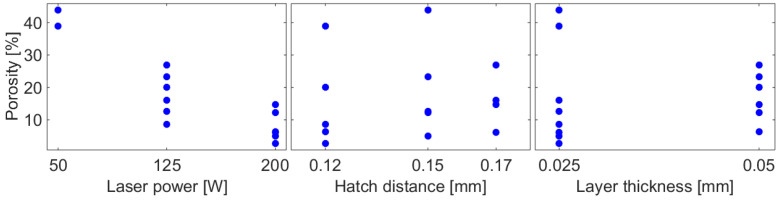
Effect of laser power, hatch distance, and layer thickness on the porosity of the cuboid samples made from Ti6Al4V alloy.

The envelope porosity measurements of the cuboid samples are shown in Figure 4. The analysis of variance demonstrated that all the investigated factors significantly impact pore formation (*p*-value ≤ 0.001). Specifically, the laser power exhibited a negative effect, while the hatch distance and the layer thickness demonstrated a weakly positive effect, detectable only when accounting for the absence of Ti15–Ti18 samples. The regression analysis finally revealed that the material porosity φ is primarily influenced by the inverse of laser power, along with the combined effect of laser power and the inverse of hatch distance and layer thickness. In fact, the most accurate model obtained from linear regression by including the reciprocal terms of the main variables and their interactions is the following:

(1) $$\varphi =11.79+\frac{1598.7}{P}-\frac{0.00035\cdot P}{h\cdot t}$$

where *P* is the laser power [W], *h* [mm] is the hatch distance, and *t* [mm] is the layer thickness. The model yields a coefficient of determination R^2^ = 0.95. Conversely, exponential models did not prove effective.

### 3.2. Surface Characteristics

**Figure 5:**
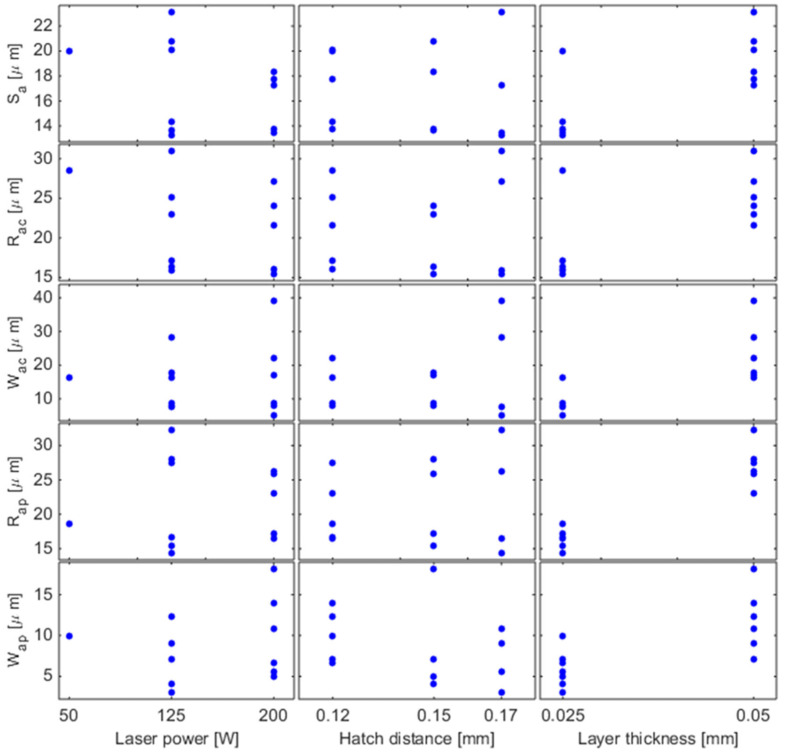
Effect of laser power, hatch distance, and layer thickness on the surface roughness (*S_a_*), linear roughness parallel to the growth direction (*R_ac_*), linear roughness parallel to the building platform (*R_ap_*), linear waviness parallel to the growth direction (*W_ac_*), and linear waviness parallel to the building platform (*W_ap_*) of the cuboid samples made from Ti6Al4V alloy.

The average surface roughness (*S_a_*), linear roughness parallel to the growth direction (*R_ac_*), linear roughness parallel to the building platform (*R_ap_*), linear waviness parallel to the growth direction (*W_ac_*), and linear waviness parallel to the building platform (*W_ap_*) of the cuboid samples are presented in Figure 5. The Ti14 sample was excluded due to its reduced size, which prevented accurate measurements. The analysis of variance demonstrated that layer thickness significantly affects all the analyzed surface characteristics (*p*-value < 0.003) with a positive effect, while laser power significantly influences both the average surface roughness and the average linear roughness parallel to the growth direction (*p*-value ≤ 0.01), with a negative effect. The regression analysis finally revealed that the most accurate models for describing the surface characteristics of the material are exponential functions proportional to layer thickness, as follows:

(2) $$S_{a}=357.8\cdot \frac{t^{0.5}}{P^{0.3}}\;[\mu m]$$

(3) $$R_{ac}=1032.8\cdot \frac{t^{0.6}}{P^{0.4}}\;[\mu m]$$

(4) $$W_{ac}=1480.3\cdot t^{1.4}\;[\mu m]$$

(5) $$R_{ap}=234.2\cdot t^{0.7}\;[\mu m]$$

(6) $$W_{ap}=19.7\cdot \frac{t^{1.1}}{h^{1.4}}\;[\mu m]$$

where *P* [W] is the laser power, *h* [mm] is the hatch distance, and *t* [mm] is the layer thickness. The models exhibit excellent descriptive power, as demonstrated by their respective coefficients of determination: ${R^{2}(S}_{a})=0.90$, ${R^{2}(R}_{ac})=0.84$, ${R^{2}(W}_{ac})=0.71$, ${R^{2}(R}_{ap})=0.88$, ${R^{2}(W}_{ap})=0.72$. Conversely, polynomial models did not prove effective.

### 3.3. Surface Morphology

**Figure 6:**
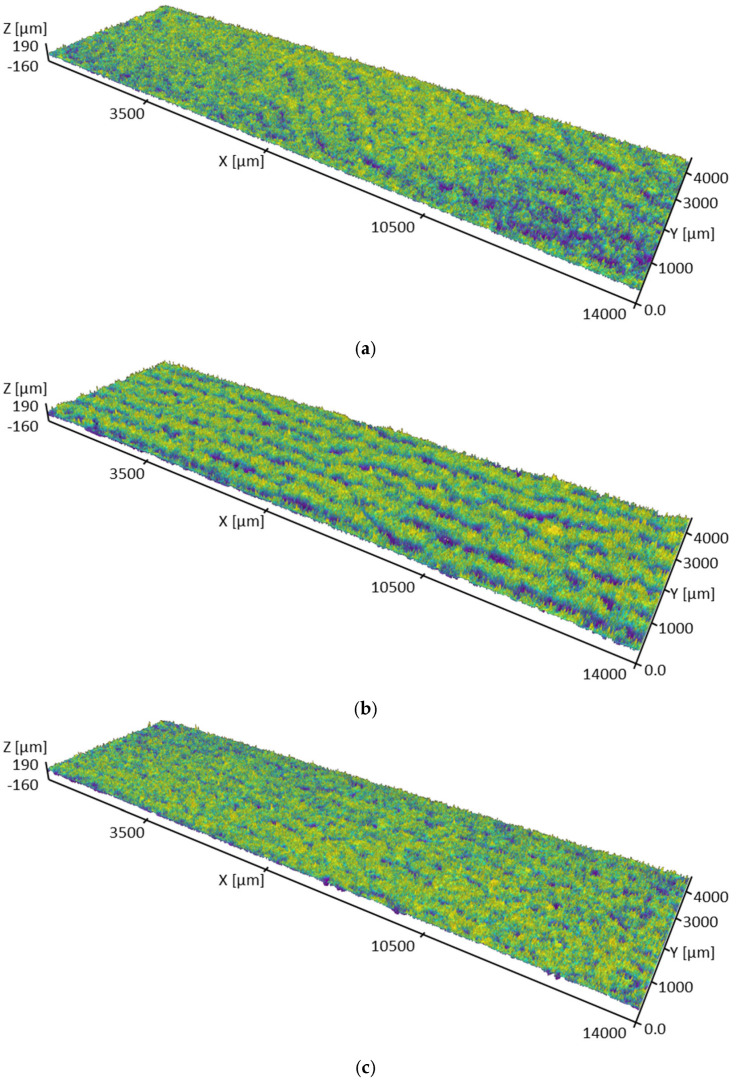

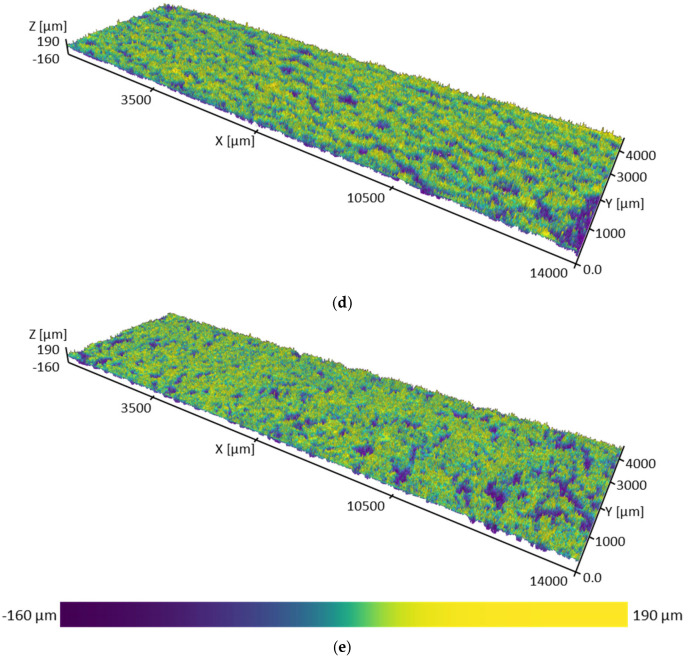
Representative examples of the surface morphology of the cuboid samples (**a**) Ti1, (**b**) Ti4, (**c**) Ti7, (**d**) Ti10, and (**e**) Ti13.

Investigating surface morphology by examining the qualitative visual features of the sample surfaces at a larger scale, rather than focusing solely on surface roughness and individual powder particle size, is crucial for understanding material properties and interpreting surface area analysis results. The confocal microscopy revealed that the surfaces of the cuboid samples exhibit prominent protrusions and localized cavities, as well as periodic undulations with wavelengths of several tenths of a millimeter. Repetitive characteristics observed across multiple samples led to the identification of five distinct morphological classes. Class 1 comprises samples Ti1, Ti2, and Ti3, which exhibited only localized defects of limited severity. Class 2 includes samples Ti4, Ti5, and Ti6, which featured distinct undulations that were faintly discernible on samples Ti7, Ti8, and Ti9 (class 3), and slightly noticeable on samples Ti10, Ti11, and Ti12 (class 4). Sample Ti13 (class 5) presented globular defects with the highest severity among all samples. Representative examples of the distinct morphological classes are depicted in Figure 6. These defects are believed to originate from the balling phenomenon, an instability of the molten pool that characteristically occurs in the low-energy-density regime. The extent of the surface defects significantly increases with increasing layer thickness and decreasing laser power, while the hatch distance showed no significant effect, suggesting that very similar surface structures can be obtained in lamellar samples. In turn, the observed morphology appears capable of promoting a significant increase in the available surface area, which is hence expected to be primarily related to the surface roughness.

### 3.4. Pore Size and Surface Area

**Figure 7:**
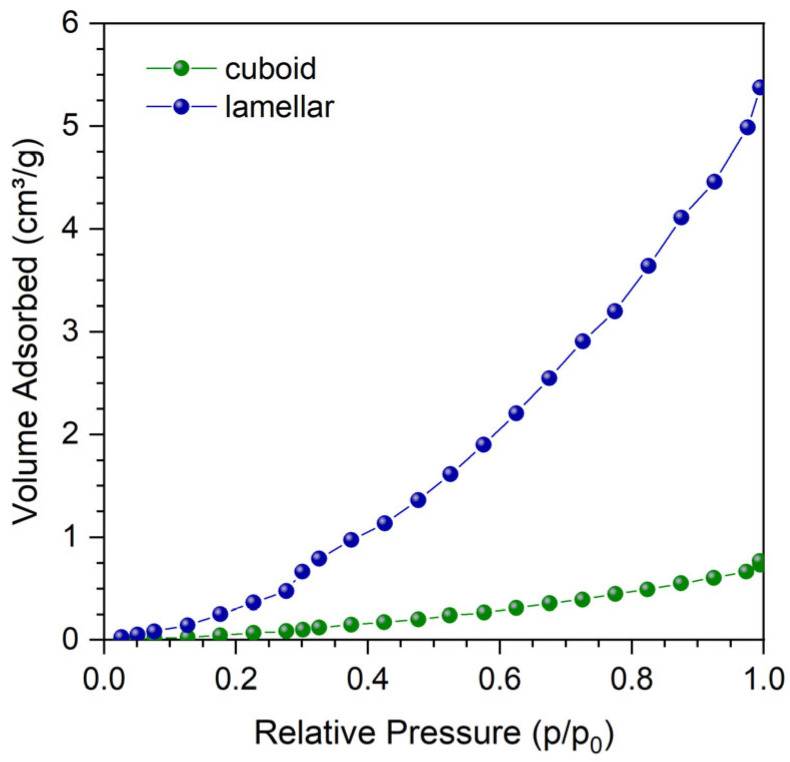
Representative adsorption branch of the N_2_ physisorption isotherms measured at 77 K on the Ti13 cuboid and lamellar sample, showing a type III isotherm [52]. The linear range for BET analysis is limited to the first 3 points.

In order to measure the porosity of prepared samples, the total surface area (in terms of m^2^/kg) and pore volume and size (in cm^3^/kg and nm, respectively) were measured by N_2_ physisorption. Surface area and pore volume values are reported in Table 3. A linear trend with the porosity values extrapolated by the envelope density analysis can be appreciated, with the samples characterized by higher porosity also exhibiting higher surface area, suggesting that the porosity control by LB-PBF parameters optimization also results in a slightly larger available surface area. However, looking at the absolute values and at the adsorption isotherms (Figure 7), which exhibit a clear type III profile in the IUPAC classification, the porosity appears to be overall closed, i.e., most of the porosity estimated by density analysis (Figure 4) is not available for any fluid to pass through the lamellar sample. Likely, the increase in available surface area is due to increased surface roughness (Figure 6). On all samples, an average pore size of around 3 nm is calculated, excluding the presence of intermediate (3–50 nm) mesoporosity and even larger (50–500 nm) macroporosity on the samples [52]. Micrometer-scale-sized open porosity, corresponding to surface roughness, can be detected by the morphology analysis in Figure 6.

Comparing the different samples’ shapes—i.e., cuboid versus lamellar—an increase of about an order of magnitude can be noticed both in the surface area and pore volume values, in line with the increased surface-to-volume ratio of lamellar samples against the cuboids. Most importantly, it can be highlighted that the linear trend observed for the cuboid samples is somewhat lost in the lamellae, suggesting that the different surface roughness/finishing and process parameters result in sensible variations in the final surface properties, which become more noticeable with increasing surface/volume ratio. This phenomenon might be exploited for the preparation of open-channeled monoliths, such as honeycombs and POCSs, where different LB-PBF process parameters, such as the ones employed for sample Ti9, would lead to a maximization of the exposed surface compared to bulk monolith samples (sample Ti13) by increasing the specific surface area of the thin channel walls while maintaining lower internal porosity and, consequently, higher mechanical resistance. Further investigation would be required to understand the peculiar properties exhibited by sample Ti9.

### 3.5. X-Ray Scattering

**Figure 8:**
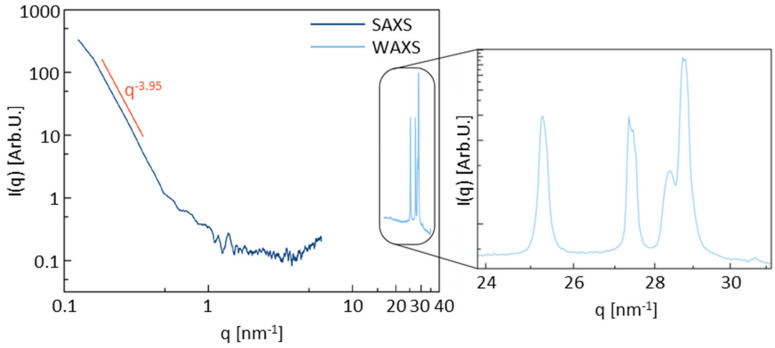
Intensity of the scattered X-ray radiation as a function of the transferred wavevector in the small-angle X-ray scattering (SAXS) and wide-angle X-ray scattering (WAXS) tests conducted on the Ti9 lamellar sample.

Small-angle X-ray scattering (SAXS) and wide-angle X-ray scattering (WAXS) analyses were conducted on the Ti9 lamellar sample, identified as the optimal configuration. The results presented in Figure 8 reveal the material’s pronounced crystallinity, as evidenced by sharp, well-defined peaks in the WAXS region. The splitting of these peaks suggests the existence of multiple crystalline phases, likely associated with various intercalants introduced during the manufacturing process.

In the SAXS region, the red-highlighted slope of the curve indicates the presence of high-electron-contrast objects with distinct spherical morphology, which likely reflect the presence of weakly sintered metal particles on the sample surface. Although the precise size and distribution of these objects remain unresolved due to experimental limitations, their characteristic size is estimated to exceed 60 nm. Additionally, a broad band of diffuse intensity appears to be present in the transition region between the SAXS and WAXS curves, suggesting a second population of high-electron-contrast objects with an estimated characteristic size of approximately 6 nm.

## 4. Conclusions

This study investigates the possibility of precisely modulating the porosity of Ti6Al4V alloy parts fabricated via laser based-powder bed fusion, with the aim of producing components featuring tailored surface and internal porosity. While this capability remains largely unexplored, it holds significant potential for advancing the technological development of topologically optimized components, implants and prosthetics, fuel production and storage devices, filtration and catalytic systems in the form of periodic open cellular structures, and other cutting-edge products. To this end, cuboid and lamellar samples were manufactured by systematically varying laser power, hatch distance, and layer thickness, and were examined to evaluate envelope porosity, surface characteristics, surface area, and surface morphology across different scales.

The experimental results demonstrated the possibility to effectively control the porosity of Ti6Al4V parts produced via LB-PBF up to approximately 44%. Laser power and layer thickness were identified as the primary factors influencing envelope porosity and surface characteristics, and accurate models describing these relationships were developed and presented. The smooth and coherent trends in the experimental data, the very low *p*-values from the analysis of variance, and the high coefficients of determination for the models indicated strong reliability and significance of the results.

N_2_ physisorption analysis revealed that the majority of the porosity generated in the samples is closed, and most of the available surface area must be attributed to surface roughness. This finding was corroborated by surface morphology analysis, as well as by small-angle X-ray scattering and wide-angle X-ray scattering tests, which suggested the presence of weakly sintered metal particles on the sample surface.

The lamellar samples exhibited an increase of nearly one order of magnitude in both surface area and pore volume, showing their potential for the production of open-channeled monolithic structures.

In conclusion, this work establishes a robust framework for tailoring porosity in Ti6Al4V parts produced via LB-PBF and provides optimal process parameters for manufacturing parts with high surface area for various industrial applications.

## Figures and Tables

**Table 1:** Chemical composition of Ti6Al4V powder.

Element	Ti	Al	V	Fe	C	H	O	N
Ti6Al4V (% weight)	Bal.	5.96	4.13	0.22	0.01	0.001	0.1	0.01

**Table 2:** Design of Experiments for optimizing the process parameters for Ti6Al4V alloy. The first column (Code) lists the identification numbers assigned to the samples, while the last row (Ref.) presents the optimal process parameters providing fully dense parts [45]. ✓ indicates conformal samples, X indicates unformed or defective samples, and - indicates unproduced samples. The scan speed and laser beam diameter were held constant at 1300 mm/s and 0.155 mm, respectively.

Code	Laser Power [W]	Hatch Distance [mm]	Layer Thickness [mm]	Cuboid Samples	Lamellar Samples
Ti1	200	0.12	0.025	✓	-
Ti2	200	0.15	0.025	✓	-
Ti3	200	0.17	0.025	✓	-
Ti4	200	0.12	0.05	✓	-
Ti5	200	0.15	0.05	✓	-
Ti6	200	0.17	0.05	✓	-
Ti7	125	0.12	0.025	✓	X
Ti8	125	0.15	0.025	✓	X
Ti9	125	0.17	0.025	✓	✓
Ti10	125	0.12	0.05	✓	✓
Ti11	125	0.15	0.05	✓	✓
Ti12	125	0.17	0.05	✓	✓
Ti13	50	0.12	0.025	✓	X
Ti14	50	0.15	0.025	✓ *	-
Ti15	50	0.17	0.025	X	-
Ti16	50	0.12	0.05	X	-
Ti17	50	0.15	0.05	X	-
Ti18	50	0.17	0.05	X	-
Ref.	225	0.09	0.025	-	-

* Conformal sample showing a strong tendency to crumble under minimal stress.

**Table 3:** Surface area and pore volume values obtained by N_2_ isotherms at 77 K for selected Ti6Al4V cuboid and lamellar samples.

Sample	Cuboid		Lamellar	
	Surface Area (m^2^/kg)	Pore Volume (cm^3^/kg)	Surface Area (m^2^/kg)	Pore Volume (cm^3^/kg)
Ti7	16	0.5	106	4
Ti8	17	0.5	151	2
Ti9	25	0.5	276	5
Ti10	22	0.5	88	3
Ti11	34	0.6	115	2
Ti12	36	0.6	147	4
Ti13	47	0.8	210	6

## Data Availability

The original contributions presented in the study are included in the article, further inquiries can be directed to the corresponding authors.
